# Role of Air-Entraining Agent in Frost Resistance and Water Absorption Prediction for Gel-Modified Coal Gangue Concrete

**DOI:** 10.3390/gels12040318

**Published:** 2026-04-08

**Authors:** Ruicong Han, Xiaoning Guo, Junfeng Guan, Min Zhang, Shuanghua He, Bin Liu

**Affiliations:** 1 School of Civil Engineering and Transportation, North China University of Water Resources and Electric Power, Zhengzhou 450045, China; hanruicong@ncwu.edu.cn (R.H.); gxn0522@163.com (X.G.); zhangmin@ncwu.edu.cn (M.Z.); liubin15503843517@163.com (B.L.); 2 School of Civil Engineering and Architecture, Guangxi University, Nanning 530004, China; 3 School of Water Conservancy, North China University of Water Resources and Electric Power, Zhengzhou 450046, China

**Keywords:** air-entraining agent, capillary water absorption, gel-modified coal gangue aggregate, frost resistance, predictive model, concrete

## Abstract

Due to the high water absorption of coal gangue aggregate, concrete prepared with a high content of this material exhibits a significantly reduced service life under freeze–thaw conditions. This study evaluates the frost resistance of gel-enhanced coal gangue aggregate concrete modified by incorporating nano-SiO_2_ and polypropylene fibre (PPF) to generate more C-S-H gel and form a dense structure with different dosages of air-entraining agent (0, 0.004%, 0.008%, 0.012%, and 0.016%). The research results show that when the admixture content is 0.012%, the concrete still exhibits excellent frost resistance after 100 freeze–thaw cycles. The mass loss is only 4.7%, compressive strength loss is 37%, and dynamic elastic modulus loss is 39%, while the specimen maintains the best apparent integrity. In addition, the capillary water absorption rate, initial capillary water absorption rate, and cumulative water absorption all reach their lowest values under this condition, indicating optimal frost resistance performance. Finally, through regression analysis, a highly accurate predictive model for capillary water absorption was established, providing a theoretical basis for further research on the durability and frost resistance of coal gangue aggregate concrete.

## 1. Introduction

Coal gangue is a type of hard rock associated with coal and a by-product of the coal mining and washing processes. It is categorized as a bulk industrial solid waste. In terms of volume, its generation accounts for about 10% to 15% of the total coal production [1,2]. The stockpiling of spontaneously combusted coal gangue emits large volumes of noxious gases such as sulfur dioxide and carbon monoxide. When used in backfilling, it occupies extensive land resources and contaminates groundwater through the leaching of heavy metals, thereby posing potential risks to human health [3]. Key industries such as chemical production, brick manufacturing, power generation, and road construction are the primary consumers of coal gangue [4]. Expanding its use in the construction industry—particularly by substituting natural gravel with coal gangue aggregate in building, pavement, and bridge engineering—plays a critical role in conserving energy and advancing green building materials [5,6,7].

**Figure 1:**
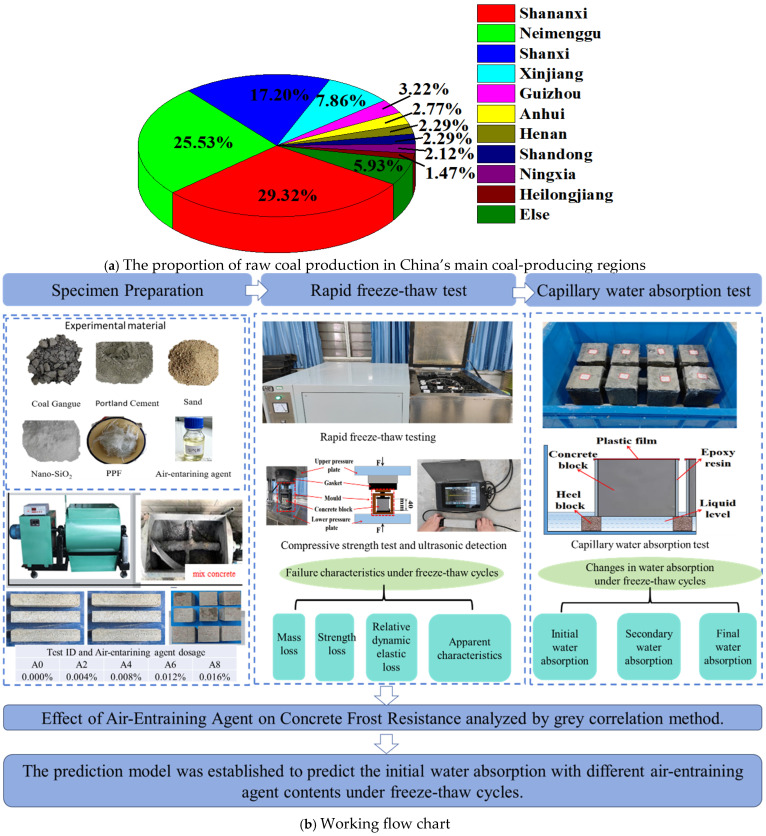
Coal gangue distribution and workflow chart.

Most of China’s coal mines are located in the cold northern regions. As illustrated in Figure 1a, the distribution of raw coal production highlights key provinces and autonomous regions such as Xinjiang, Shanxi, Shaanxi, and Inner Mongolia. Given the local availability of raw materials, a critical constraint in the widespread adoption of coal gangue is whether its frost resistance satisfies practical engineering requirements [8,9].

A number of studies have been conducted to evaluate the mechanical properties of coal gangue aggregate concrete under freeze–thaw conditions. Among them, Guan et al. [10,11] investigated the durability and frost resistance of this material, demonstrating that the degradation of its physical and mechanical properties accelerates with increasing coal gangue content. The freeze–thaw cycles led to an increase in the concrete’s peak strain and damaged layer thickness, which was accompanied by a significant reduction in the corresponding initial elastic modulus, peak stress, fracture toughness, and fracture energy. Gao et al. [12,13] developed a non-dimensional model (x = ε/ε_c_, y = σ/σ_c_) to represent the stress–strain relationship of coal gangue concrete after freeze–thaw cycles, accounting for the influence of the number of cycles and the rate of coal gangue replacement. The experimental data was quite well-fitted by this model. Qiu et al. [14] assessed the damage caused by freeze–thaw to coal gangue concrete and developed an evolution model for the damage. It was shown that as the coal gangue concentration rose, the relative mass, compressive strength, and relative dynamic elastic modulus quickly declined. The highest replacement ratio of coal gangue concrete in cold regions was 40%. Qiu et al. [15] investigated the microscopic morphology and pore structure degradation of coal gangue concrete and presented a quantifiable link between pore structure and strength degradation. Increasing the amount of admixtures results in a decrease in compressive strength and ultrasonic velocity. Cao et al. [16] conducted a study on the freeze resistance of recycled aggregates and found that when the number of freeze–thaw cycles reached 25, the appearance of the recycled concrete specimens was compared with that of the ordinary concrete specimens before the freeze–thaw process. The cement slurry on the surface of the specimens only showed slight detachment, and the degree of detachment was similar for both. As the number of freeze–thaw cycles increased, the phenomenon of mortar detachment on the surface of the specimens became more obvious, and this phenomenon became more pronounced with each additional cycle. After 100 freeze–thaw cycles, the appearance characteristics of the recycled concrete specimens showed more severe damage compared to the ordinary concrete specimens. However, by refining the pore structure—particularly by increasing the content of micropores smaller than 0.3 mm—the freeze–thaw resistance is improved. Furthermore, the quantitative stereological parameters offer strong predictive evidence for the behaviour of air-entrained concrete, as investigated by Zalegowski [17]. They discovered that while a small amount of glycol could increase the salt freeze resistance of concrete, the influence of salt freeze–thaw erosion decreased with an increase in inorganic antifreeze.

The aforementioned research findings indicate that the main factor limiting the applicability of full coal gangue concrete is frost resistance, which leads to the reduced service life of structures. Thus, there are a lot of opportunities for application in the study of improving the frost resistance of all coal gangue aggregate concrete. Concrete containing full-coal gangue aggregate and having a C25 strength rating has previously been produced [18]. However, no research on the freeze resistance performance of full coal gangue concrete has been conducted. Therefore, in this study, different concentrations of air-entraining agents were applied to coal gangue concrete to assess the impact on the concrete’s ability to withstand frost and absorb water through its capillaries. The working flow chart is shown in Figure 1b. The dosages of air-entraining admixture that were chosen as 0%, 0.004%, 0.008%, 0.012%, and 0.016%. Furthermore, after undergoing 0, 25, 50, 75, and 100 freeze–thaw cycles, the quality loss, apparent damage, strength damage, and dynamic elastic modulus of the specimens were tested. In addition, the capillary water absorption performance of concrete at different freeze–thaw stages was further tested. The influence of an air-entraining agent on the anti-freezing performance was evaluated by using the grey theory. Finally, a predictive model for capillary water absorption in the initial stage was established. At the same time, it provides a strong theoretical basis for the future engineering application of full coal gangue concrete, such as highways and low-rise buildings.

## 2. Results and Discussion

### 2.1. Surface Mass Loss

**Figure 2:**
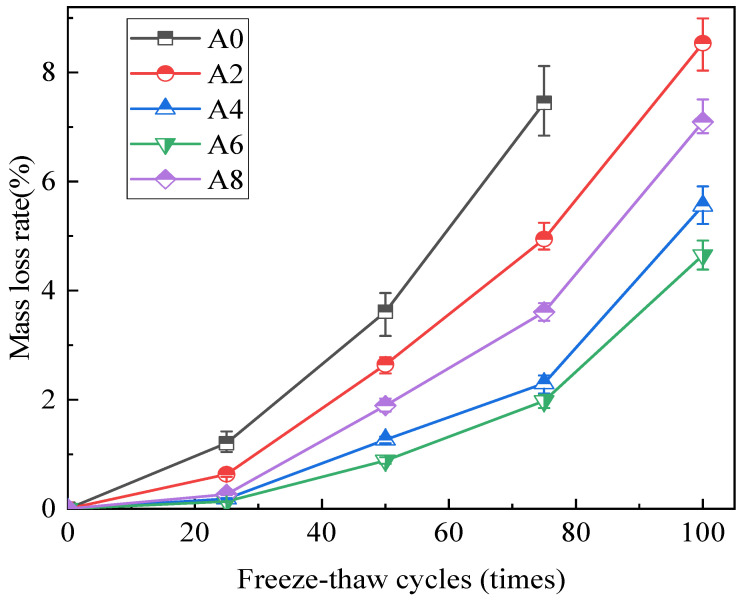
The quality loss rate of coal gangue concrete under different freeze–thaw cycles.

The impact of the number of freeze–thaw cycles on the concrete mass loss rate is shown in Figure 2. The test results show an increase in the mass loss rate of the concrete specimen. Groups A2 and A4 saw greater rates of mass loss through 25 freeze–thaw cycles. After 50 freeze–thaw cycles, the mass loss rate of concrete increases significantly. Group A0 had a mass loss rate of 7.44%, Group A2 had a mass-loss rate of 4.95%, and the other three groups had very minor mass losses after 75 freeze–thaw cycles. Group A6 had a loss ratio of just 4.65%, whereas groups A2, A4, and A8 had loss ratios of 8.54%, 5.56%, and 7.10%, respectively. Compared to the work of Gao et al. [13], who used 60% coal gangue in place of coarse aggregate, the mass loss after 100 freeze–thaw cycles was 5.43%, and the inclusion of an air-entraining agent significantly increased the coal gangue concrete’s resistance to cold. However, excessive admixture content results in the formation of too many independent bubbles in the concrete with extremely close spacing. When hydration and shrinkage occur, shrinkage cracks are likely to penetrate these independent pores, forming interconnected pores that allow water to enter and induce frost expansion [19].

The weakest part of coal aggregate concrete is the interfacial transition zone. Concrete’s ability to withstand freeze–thaw damage is rapidly declining as a result of the increased frequency of freeze–thaw cycles, which also causes new and existing cracks in the interfacial transition zone to appear and spread. This accelerates the degradation of freeze–thaw damage and highlights quality loss [20]. As a result, after 50 cycles of testing, the mass loss of concrete without an air-entraining agent has reached 5%. Based on the research results of concrete with 50% of the coarse coal gangue aggregate replaced separately, after 100 freeze–thaw cycles, the quality loss of coal gangue concrete was also within the range of 4% to 5% [21], demonstrating that the concrete specimen’s mass loss rate can be decreased and the expansion force caused by freeze–thaw can be efficiently mitigated by the airtight bubbles created by the air entrainment agent.

### 2.2. Apparent Damage Characteristics

**Figure 3:**
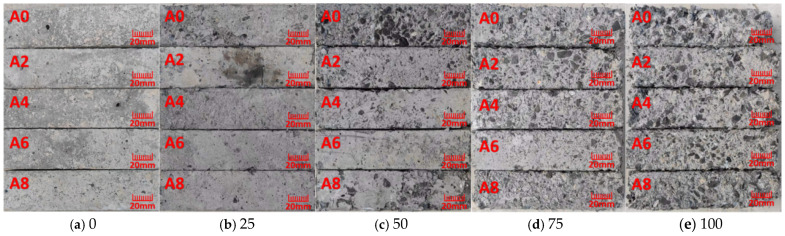
Appearance of coal gangue concrete under different freeze–thaw cycles.

The most significant difference between coal gangue and natural aggregate is the different water absorption rate, which has a certain influence on the strength and frost resistance of concrete. Figure 3 displays the samples’ morphology after freeze–thaw testing. When they were tested after 25 times, small pores of A0 and A2 of the test specimens increased, and specimens appeared rough, while there was no change in the other three groups. When the number of freeze–thaw cycles reached 50, the mortar on the surface of A0 was completely peeled off, the aggregate was exposed, the surface of specimens A2, A4 and A8 was spalling and pitting, and the surface of specimen A6 became rough. After 75 continuous freeze–thaw cycles, the aggregate of specimen A0 was spalling, the edges were broken, and the whole was loosened; the surface mortar of A2, A4, and A8 was completely spalling, and the aggregate was exposed, while the phenomenon of large holes and pit erosion began to appear in A6. After 100 freeze–thaw cycles, group A6 was relatively intact in terms of surface mortar spalling. The edges and corners of other four groups were all damaged. The samples’ freeze–thaw damage may be efficiently slowed down by adding an air-entraining agent, as demonstrated by the findings, which also indicates that the apparent damage trend is consistent with the mass loss data. Qiu et al. [14] conducted a study where 60% of the coarse coal gangue was replaced and subjected to 100 freeze–thaw cycles. The results indicated that phenomena such as large pores and pitting occurred, and some aggregates were exposed. This was consistent with the phenomenon observed in Group A6 of this paper. This shows that the air-entraining agent has a significant improvement effect on the freeze resistance of full coal gangue concrete.

### 2.3. Loss of Strength

**Figure 4:**
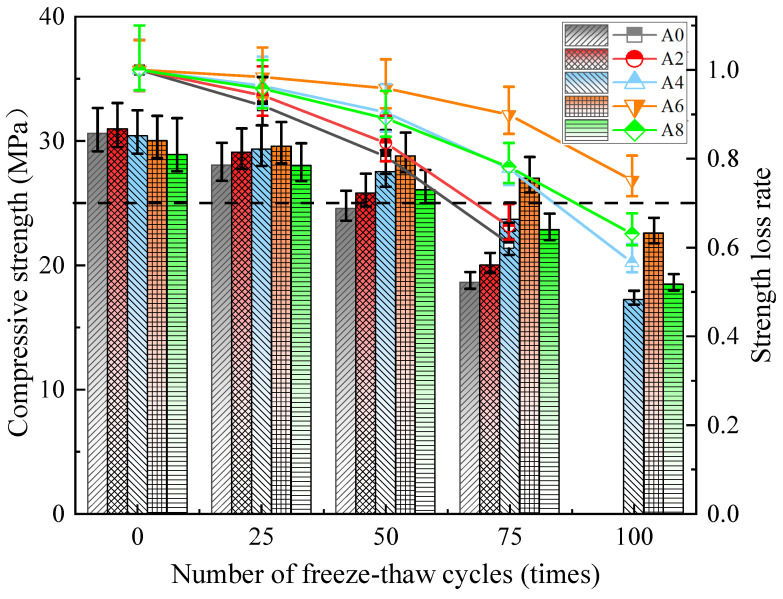
Strength and strength loss rate of coal gangue concrete under different freeze–thaw cycles.

The impact of an air-entrainment agent on the compressive strength of concrete during a freeze–thaw cycle is seen in Figure 4. When there was no freezing and thawing, concrete’s compressive strength initially increased and subsequently fell in accordance with the rate at which the air entrainment agent was replaced. The concrete in Group A2 had the greatest compressive strength of all of them, at 30.87 MPa. This is because concrete may become more hydrated and more fluid when a small quantity of air entrainment agent is added to it. However, too many pores might be harmful.

With an increasing number of freeze–thaw cycles, the compressive strength of all the specimens decreased. The five sets of specimens’ compressive strengths did not considerably decline before 25 freeze–thaw cycles. Rapid strength reduction can be seen after 50 freeze–thaw cycles, especially for Group A0 and Group A2. This is the initial stages of freeze–thaw, a significant amount of unhydrated cement particles were present in the concrete, and some new hydrated cementing materials will also be repaired during this time [22]. Under the same test conditions, concrete exhibits low strength, a high strength loss rate, and poor freezing resistance when the air-entraining agent is either absent or present in modest amounts. The compressive strength of the A0, A2, A4, and A8 specimens decreased by 39.0%, 35.2%, 22.2%, and 21.8%, respectively, when the number of tests reached 75. The other four groups, with the exception of Group A6, have reached the failure condition (the strength losses of A0 and A2 exceeded 25%). The specimen’s pore wall experiences pressure as the free water inside it freezes and expands, which leads to an expansion failure of the coal gangue concrete and more internal damage. The concrete strengths of A4, A6, and A8 dropped by 44.4%, 24.9%, and 36.9%, respectively, after being continuously exposed to 100 freeze–thaw cycles (the strength losses of A4 and A8 exceeded 25%). Furthermore, 0.012% (Group A6) was the optimal dosage for anti-freezing performance. However, when the dosage reached 0.016% (Group A8), the performance declined. The main reason for this was that excessive air-entraining agent caused an increase in pores and a shortening of the distance between bubbles, making it easier for interconnected cracks to form.

The results show that air-entraining agents can effectively improve the frost resistance of coal gangue aggregate concrete. Coal gangue concrete’s resistance to frost is increased when air-entraining agents are used because they can create a large number of homogeneous, airtight bubbles inside the concrete, divide the capillary channel inside the concrete, and buffer the expansion pressure caused by concrete freeze–thaw and the osmotic pressure of capillary water. However, excessive addition of air-entraining agents leads to excessive increase in pores, which has a negative effect. Furthermore, compared with the latest research results [23], when 45% of coal gangue is added, after 100 freeze–thaw cycles, the compressive strength loss of the coal gangue concrete reaches more than 25%, which fully indicates that the addition of air-entraining agent can significantly effectively improve the freeze resistance of coal gangue concrete.

### 2.4. Relative Dynamic Elastic Modulus

**Figure 5:**
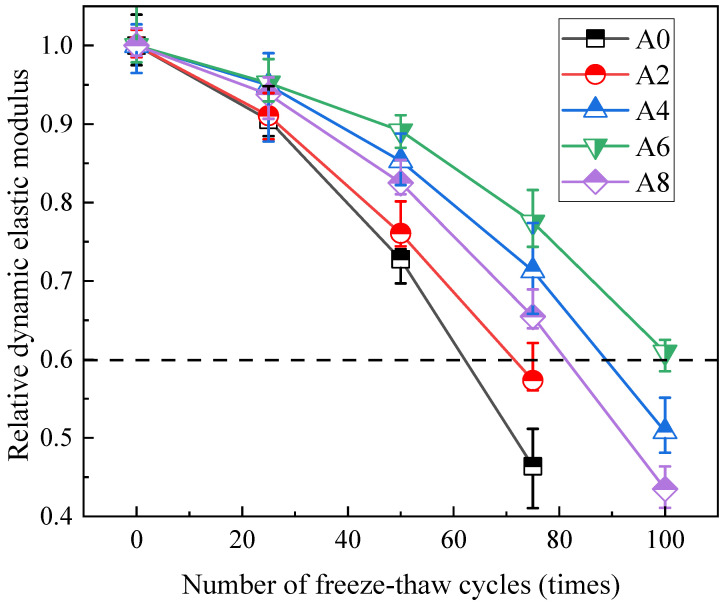
Relative dynamic elastic modulus of coal gangue concrete variation curve of freeze–thaw cycles.

The relative dynamic elastic modulus indicates the degree of deterioration of coal gangue concrete specimens. Figure 5 illustrates how the relative dynamic elastic modulus of coal gangue concrete changes under freeze–thaw conditions. Every specimen experiences a steady drop in relative dynamic modulus as the number of test cycles increase. A faster aggregate water absorption rate is one of the primary causes of the acceleration of freeze–thaw failure in concrete. This is consistent with the causes of the aforementioned damage characteristics and strength loss characteristics. The relative dynamic elastic modulus for Group A0 and Group A2 decreased to 0.45 and 0.56 after 75 cycles. The relative dynamic modulus of A4 and A8 dropped to 0.51 and 0.43, respectively, after 100 consecutive tests, whereas that of A6 declined to 0.6,1 and the relative dynamic modulus loss did not surpass 40%. Zhang et al. [24] added anhydroethylene glycol antifreeze and discovered that the concrete became damaged after 60 freeze–thaw cycles, demonstrating that the air-entraining agent’s antifreeze performance is superior to that of the antifreeze agent.

Compared with the latest research, under similar water–binder ratio conditions, replacing 40% coarse coal gangue aggregate and 80% fine coal gangue aggregate [25], or only replacing 100% coarse coal gangue aggregate [26], the dynamic elastic modulus loss of concrete after 100 freeze–thaw cycles is approximately 0.48–0.48. However, the addition of 0.012% air-entraining agent, as suggested in this paper, can counteract the negative impact of coal gangue on concrete to some extent.

### 2.5. Capillary Water Absorption Performance

One major factor contributing to the deterioration of concrete performance and inadequate structural durability is the production or penetration of microcracks in the concrete, which speeds up the entry of water and other media. The capillary water absorption characteristics of building materials are currently commonly employed to describe the water absorption characteristics of concrete, and they have progressively grown in importance as a means of assessing the material’s durability [27,28].

#### 2.5.1. Cumulative Water Absorption Rate

**Figure 6:**
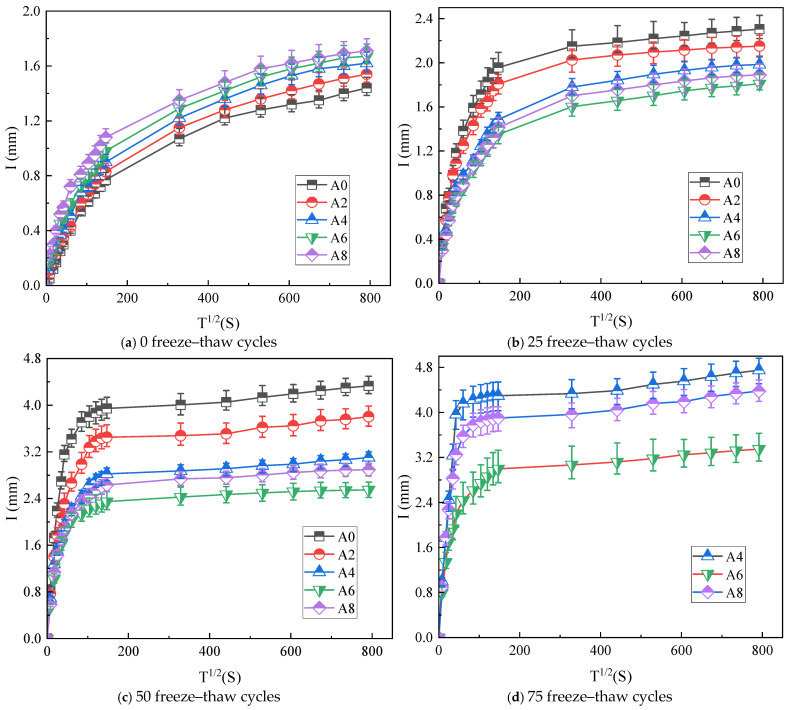
Change curve of cumulative water absorption and time square root.

Figure 6 displays the cumulative water absorption and time square root variation curves for five groups of specimens subjected to freeze–thaw cycles. The cumulative water absorption curve of concrete exhibits a nonlinear upward trend. The whole growth process may be generally split into three stages: relatively quick development occurred in the early stage (T^1/2^ = 0~147 s), sluggish growth occurred in the middle period (T^1/2^ = 147~518 s), and the latter phase (T^1/2^ = 518~831 s) tended to stay steady until the equilibrium. This is because the coal gangue aggregate concrete specimen contains numerous dispersed pores both inside and outside of it, and the coal gangue absorbs a lot of water. When unsaturated concrete comes into touch with water, the gradient between its exterior and internal forms widens, and water swiftly permeates the specimen’s surface pores, filling and transferring the interior. Over time, the water absorption curve gradually tends to become smooth, which occurs due to the fact that the hydration of the specimen increases the friction between water molecules and the wall surface of the capillary, which hinders the transfer of water [29].

The cumulative water absorption relationship (A0 < A2 < A4 < A6 < A8) of the specimens with various air-entraining agents before freeze–thaw is depicted in Figure 6a. Increased porosity was the outcome of the air-entraining chemicals’ inclusion, which created a lot of bubbles inside the concrete. On the other hand, Figure 6b–d displays the cumulative water absorption changes in the five test block groups as A6 < A8 < A4 < A2 < A0 following the freeze–thaw cycles. At 25 and 50 cycles, A0 and A2 exhibit a stronger increasing trend, whereas A0 exhibits a more noticeable upward trend with the greatest water absorption rate. A4, A6, and A8 exhibit minimal variations in the rate of water absorption. The concrete test specimens A0 and A2 were damaged when the number of cycles reached 75, while Group A6 had the lowest water absorption. Meanwhile, the rates of water absorption for A4 and A8 rose. Freeze–thaw cycles break down the thick internal structure and create more permeable pathways by increasing the porosity and damaging pores in gangue aggregate concrete. Concrete’s permeability is greatly increased by the damage. However, the distinctive pattern of particle size distribution can be enhanced by holes formed through artificial air-entrainment, hence decreasing the capillary absorption coefficient [30]. However, excessive air intake could significantly increase the specimen’s interior porosity and decrease its frost resistance.

#### 2.5.2. Water Absorption at Each Stage

**Figure 7:**
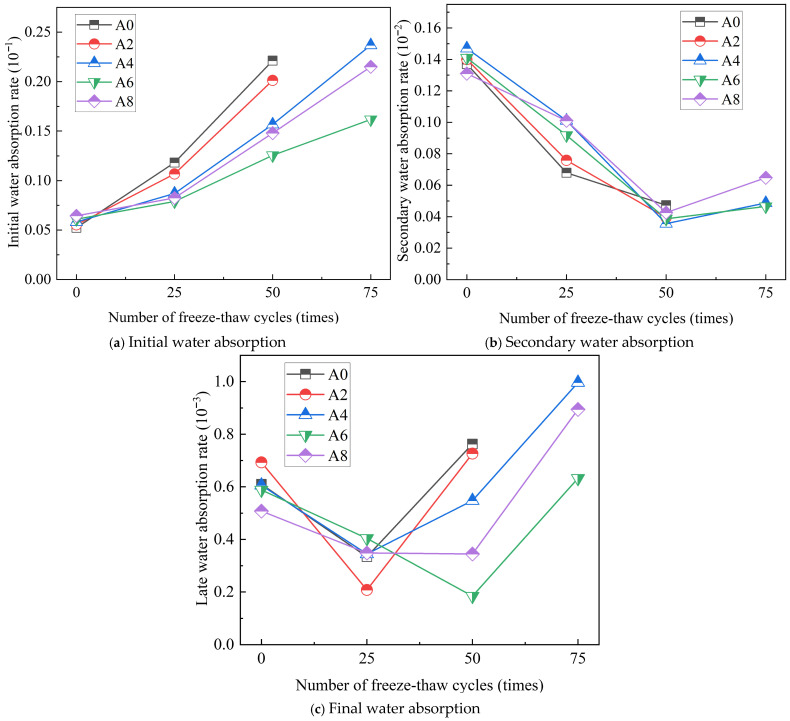
Relationship curves of water absorption at different stages under freeze–thaw cycles.

Equation (4) was used to conduct linear fitting on the first, second, and third stage curves in Figure 6 to determine the connection between each stage and the total number of freeze–thaw cycles for each of the five groups of specimens. Figure 7 illustrates the rates of primary, secondary, and ultimate water absorption that were determined.

Figure 7a illustrates how the initial water absorption of gangue aggregate concrete rises as the number of cycles increases. The initial water absorption in A0 was the greatest, and it increased 4.22 times compared to the pre-freeze–thaw period, when cycles were raised to 50. The A0 and A2 specimens were damaged after 75 testing cycles; A4 had the highest initial water absorption and increased 4.07 times, while A6 had the lowest initial absorption and increased 2.65 times. Before the test, the addition of an air-entrainment agent increased the capillary water absorption of concrete. Following the freeze–thaw cycles, the test specimens’ water absorption rate first dropped and then rose in proportion to the amount of air-entraining agent. Because A6 had the least internal damage, it may be more resistant to freeze–thaw damage.

The data in Figure 7b indicates a negative correlation between the increase in freeze–thaw cycles and the change in secondary water absorption of the concrete test specimens. This is because coal gangue aggregates have a high porosity and high water absorption rate, and, early on, capillary water absorption occurs rather quickly. As the time passes, the test specimens’ natural rate of water absorption progressively achieves saturation, which causes the rate of water absorption to significantly decline and become relatively sluggish in the test specimen’s later years [31]. The absorption of water by concrete is a slow process; water initially absorbed through the surface pores gradually migrates inward. During freeze–thaw cycles, the freezing expansion within the smaller internal pores generates microcracks, which accelerate water transport and facilitate the early filling of these pores. As a result, the number of pores available for subsequent water absorption decreases, leading to a reduction in the secondary water absorption rate. A0 and A2 exhibited much lower water absorption rates than the other three groups at 25 freeze–thaw cycles. A0 and A2 had greater surface porosity after 25 freeze–thaw cycles, which allowed the aggregate to absorb more water and lower the secondary water absorption rate. The surfaces of the remaining three sets of concrete started to spill after 50 freeze-thaw cycles, with a notable increase in the initial water absorption rate and a decrease in secondary water absorption.

Test cycles affect the ultimate water absorption, as Figure 7c illustrates, but the total change in the initial and secondary water absorption is minimal. The results indicate that, as the number of cycles increases, the capillary water absorption of the five concrete groups gradually stabilizes in the later period, remains constant once it reaches a certain value, and eventually flattens the water absorption curve.

It is evident from the study results above that the freeze–thaw cycle quickens the damage that occurs inside the test specimens. In addition, Qiu et al. [32] indicated that the initial capillary water absorption rate would rise as the number of freeze–thaw cycles increase. Furthermore, an increase in the cycle period led to a progressive rise in the specimen’s water infiltration capacity and initial water absorption rate, ultimately speeding up the destruction of concrete. By adding an air-entraining agent, concrete may be made more resistant to frost, insulated with closed air bubbles, less susceptible to internal frost heaving damage, and exhibiting a decreased rate of capillary water absorption. Furthermore, by comparison with the literature [33], the relationship between different proportions of coal gangue and capillary water absorption rate was studied. Replacing 60% of the coarse coal gangue with cement can make the capillary water absorption rate similar to that of Group A6 after 25 freeze–thaw cycles. Consequently, the use of an air-entraining agent can be regarded as a means of increasing the anti-freezing durability of coal gangue aggregate when utilized in cold places.

### 2.6. Evaluation of Frost Resistance

**Figure 8:**
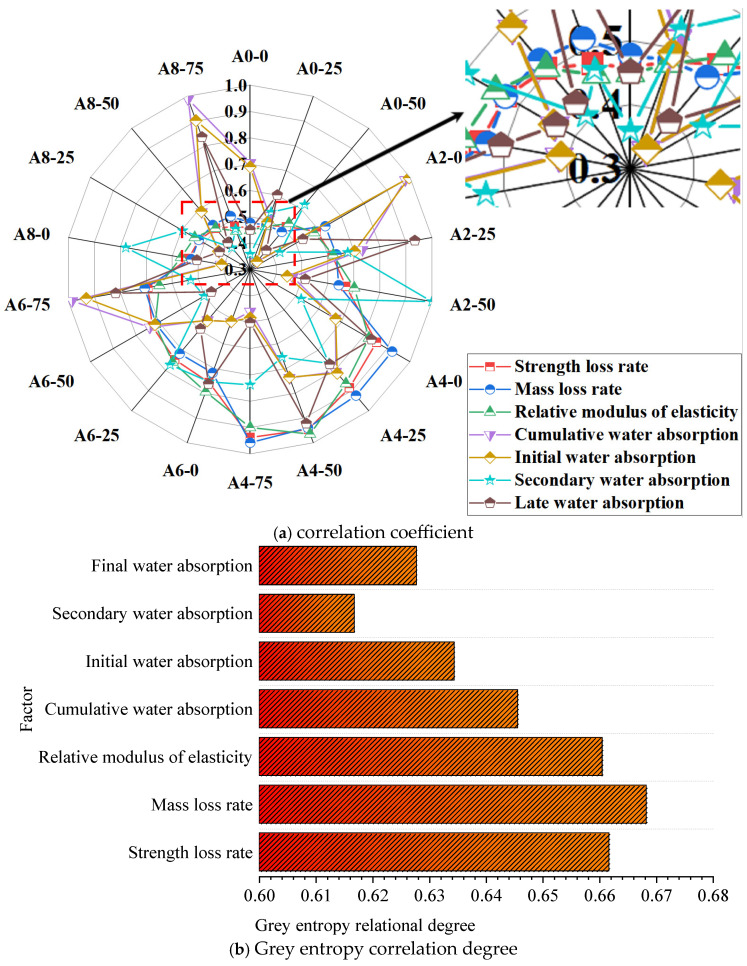
Results of grey relational entropy analysis.

The grey correlation degree was used to determine the effect of air-entraining agent concentration on coal gangue aggregate concrete’s frost resistance. The analytical index set consisted of seven items: the rate of compressive strength loss, the rate of mass loss, the relative elastic modulus, the cumulative water absorption rate, and the rates of initial, secondary, and final water absorption. The investigation of the grey correlation entropy and the correlation coefficients for various air entrainment contents and cycles are displayed in Figure 8. The correlation coefficients of the seven measures are somewhat discontinuous; yet, Figure 8b demonstrates that all seven indices have correlation degrees greater than 0.6, suggesting strong analytical outcomes. This indicates that the seven frost resistance indices are susceptible to varying degrees of influence from the air-entraining agent, and the correlation values may be used to determine the indices’ relative importance. The results show that the influencing factors of the admixture dosage on the indicators, from highest to lowest, are mass loss rate, compressive strength loss rate, relative dynamic modulus, cumulative absorption of capillary water, initial water absorption rate, maximum water absorption rate, and secondary water absorption rate.

The grey correlation degree principle states that an increase in air-entraining agent amount would result in the creation of more artificial pores in the cement matrix and that the concrete mass loss rate is most sensitive to air-entraining agent content. These pores negatively impact the other six indicators but not the mass loss rate before the freeze–thaw cycle. For instance, an increase in air-entraining agents increases the total amount of water absorbed by capillaries. In addition, the use of an air-entraining agent causes bubbles to develop within the concrete, blocking its capillary channels, reducing the expansion pressure caused by freezing and thawing, and preserving its quality.

### 2.7. Establishment of Initial Water Absorption Prediction Model

The grey correlation value of the initial water absorption rate, which is greater than 0.6, can be used to determine the capillary water absorption capacity of coal gangue aggregate concrete. Consequently, the prediction model can be considered to predict the initial water absorption with different air-entraining agent contents under freeze–thaw cycles [8,27,28,34,35].

Assumption:

(1) The process of absorbing water is one-dimensional; it ignores the effects of evaporation, chemical reactions, and other interactions between the water and its constituent elements.

(2) The concrete specimen is initially maintained in a fully dry state, and the boundary conditions remained constant throughout the entire water absorption procedure.

(3) The initial absorption of capillary water is determined by the number of freeze–thaw cycles; other environmental parameters, such as temperature during the freeze–thaw cycle, are not considered.

(4) The initial capillary water absorption increases and remains positive with an increase in freeze–thaw cycles.

#### 2.7.1. Establishment of Prediction Model

After fitting the data in Figure 7a to create the initial capillary water absorption prediction model of coal gangue concrete, it was discovered that both the cycle times and changes in the initial capillary water absorption followed an exponential distribution. In addition, the experimental results of references [31,34,36] show that the high porosity of recycled aggregate itself leads to an exponential distribution of the initial water absorption of the produced concrete. As a result, Equation (1) provides the fundamental structure of the gangue concrete water absorption prediction model:

(1) $$S_{1}\;=\;A\;\times \;B^{N}$$

where A and B are the effect coefficients of the air-entraining agent concentration, R^2^ is the regression coefficient, N is the number of freeze–thaw cycles, S1 is the starting absorption of capillary water, and Table 1 shows the fitting parameters.

Regression analysis was performed using the fitting parameters listed above, which corresponded to the dosage of air-entraining agent in each of the five specimen groups. The relationship between the air-entraining agent’s influence coefficient and replacement rate was as follows:

(2) $$A\;=\;e^{\left(-2.9-0.1x+12.4x^{2}\right)}$$

(3) B=1 − 0.18x

where x is the air-entraining agent content.

Equations (2) and (3) can be substituted into Equation (1) to get the first water absorption prediction model of gangue aggregate concrete based on the study presented above. Here is the prediction model:

(4) $$S_{1\;}={\;e}^{\left(-2.9-0.1x+12.4x^{2}\right)}\;\times \;{\left(1\;-\;0.18x\right)}^{N}$$

#### 2.7.2. Validation of Prediction Model

**Figure 9:**
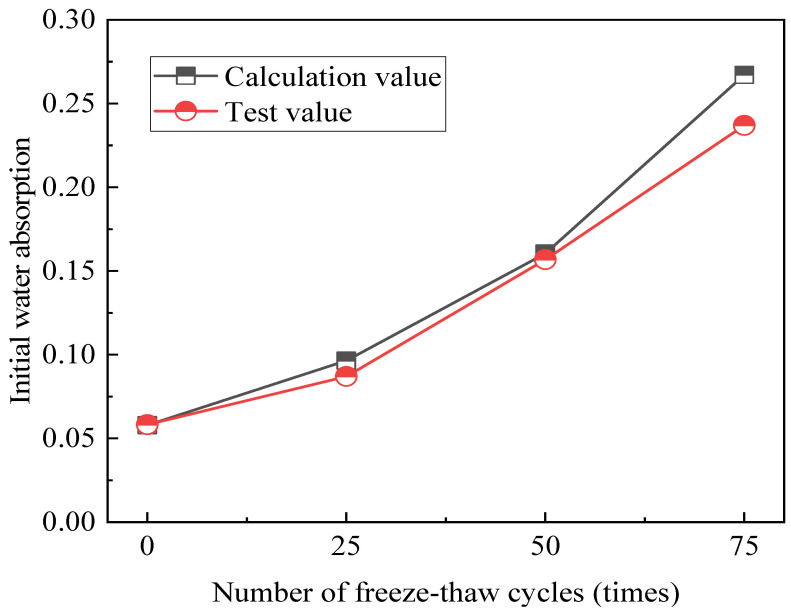
Relationship between test values and calculation values.

Using the test data from Group A4, the preliminary capillary water absorption prediction model created in this research is verified based on the test results. The entrainment content of the specimen in Group A4 was substituted into the prediction model to get the estimated values of the first capillary water absorption at various freeze–thaw durations. Then, a comparison is made using the initial capillary water absorption test data for A4 under various freeze–thaw conditions. Figure 9 displays the comparison findings. The model’s estimated values and the growth patterns of the initial capillary water absorption test values in Group A4 are similar. The results of the initial capillary water absorption test for A4 are displayed in Table 2 along with the values that were computed for the projected models in various freeze–thaw scenarios. The initial capillary water absorption test value and the computed value of the model in Group A4 have a mean squared error of 0.000495 and a root mean squared error of 0.0222, respectively. Additionally, the coefficient of determination, R^2^, is 0.948224. Table 1 and Table 2 demonstrate the great accuracy of the prediction model.

## 3. Conclusions

This study evaluates how air-entraining agents enhance the freeze–thaw resistance of a coal gangue concrete designed to generate more C-S-H gel via nano-SiO_2_ and PPF modification. The performance of the modified mixture was assessed, and the following conclusions were derived from the findings:

(1) Due to their high water absorption, coal gangue aggregates lead to poor frost resistance in concrete modified with nano-silica and PPF. The addition of an air-entraining agent modifies this property, resulting in a trend where frost resistance first increases and then decreases. At an optimal dosage of 0.012%, the concrete exhibited effective resistance to 100 freeze–thaw cycles. The corresponding losses were limited to 4.7% in mass, 37% in compressive strength, and 39% in dynamic elastic modulus, while the specimen maintained the best apparent integrity. Furthermore, the addition of 0.012% air-entraining agent achieved the optimal frost resistance, with the concrete withstanding up to 100 freeze–thaw cycles.

(2) The cumulative water absorption curve of coal gangue aggregate concrete exhibits a three-stage linear growth pattern. After freeze–thaw cycles, the initial capillary water absorption stage follows an exponential trend, the secondary stage shows an overall quadratic decline, and the later stage remains relatively stable. Notably, the concrete incorporating 0.012% air-entraining agent displays a capillary water absorption rate of only 3.3 mm, indicating the best frost resistance performance.

(3) Based on the grey correlation analysis, the addition of an air-entraining agent can effectively enhance the frost resistance of full coal gangue aggregate concrete. The correlation degrees between the frost resistance indicators of the concrete and the dosage of air-entraining agent all exceed 0.6. Among them, the mass loss rate of concrete is the most sensitive indicator reflecting the effect of air-entraining agent dosage on the improvement of frost resistance.

(4) Based on experimental results, an exponential model was established to predict the initial water absorption rate of coal gangue aggregate concrete under freeze–thaw damage, using the air-entraining agent dosage as the variable. The model shows excellent agreement with the test data, with a variance of only 0.0098. It can serve as a useful reference for studying the freeze resistance performance of such concrete within 100 cycles.

## 4. Materials and Methods

### 4.1. Experimental Material

#### 4.1.1. Cementitious Materials

The fineness of the 42.5 plain Portland cement (P.O.42.5 R) utilized in this investigation was 0.0025 mm. According to Chinese code GBT 8074-2008 [37], it was discovered that the cement had a required surface area of 342 m^2^/kg. The fly ash is made using premium fly ash (Shengtong Mineral, Shijiazhuang City, China), which has a density of 2.45 g/cm^3^ and a particle size range of 0.005–0.05 mm. The chemical makeup and performance indicators of cement and fly ash are shown in Table 3, and laboratory tap water is used as the water source.

#### 4.1.2. Coal Gangue Aggregate

**Figure 10:**
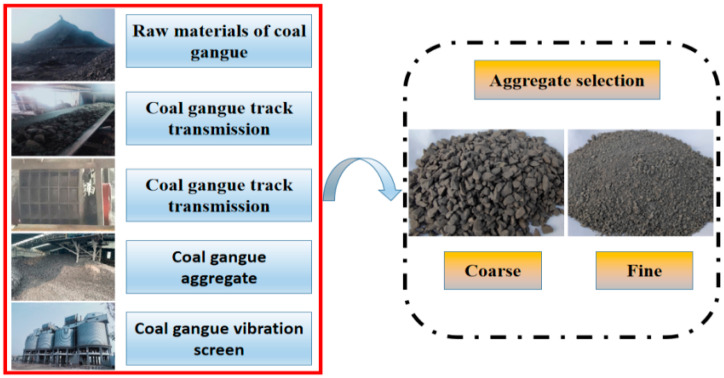
The breaking process for coal gangue with counterattack crusher.

**Figure 11:**
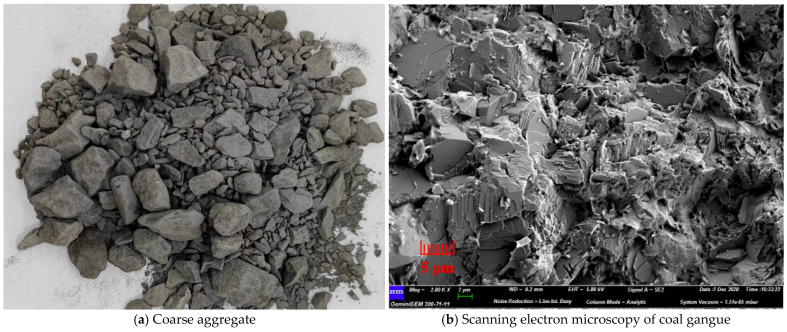

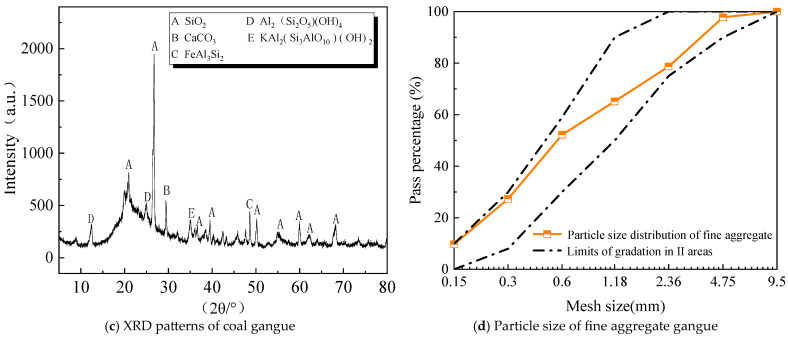
Appearance, mineral composition, and particle size distribution of aggregates.

Figure 10 illustrates the production process of coal gangue. The coarse and fine aggregates are chosen using the coal gangue generated in the Hebi mining region of Henan Province. Two kinds of coal gangue aggregates with the size of particles of 0–5 mm (fine aggregate) and 5–10 mm (corse aggregate) were prepared, and the original coal gangue was shattered by an impact crusher and then filtered using a vibrating screen. In Figure 11, the prepared aggregates are displayed. Scanning electron microscopy can detect a high number of pores on the surface of coal gangue and cracks left after crushing (as shown in Figure 11b). Figure 11a clearly shows that coal gangue has numerous corners and dust on its surface. Additionally, Table 4 indicates physical characteristics for coarse and fine coal gangue, showing that the material has a small specific surface area, a high rate of water absorption, and a big crushing value. Figure 11c displays the analysis of the mineral content. It is a composite rock made up of several different minerals, with over 70% of the composition made up of quartz and kaolinite, and only around 22% of illite. There are fewer other rocks (less than 10%). The fine aggregate utilized in the experiment has a fineness modulus of 2.45. The sand grain grading curve is depicted in Figure 11d; it has a decent particle size distribution, and it is within the top and lower bounds.

#### 4.1.3. Nano-SiO_2_ and PPF (Polypropylene Fibre)

**Figure 12:**
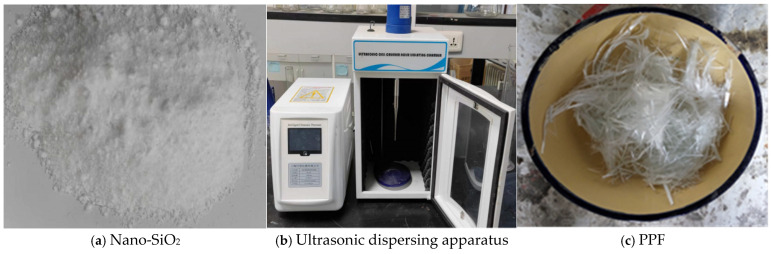
Modified materials and dispersing devices.

**Figure 13:**
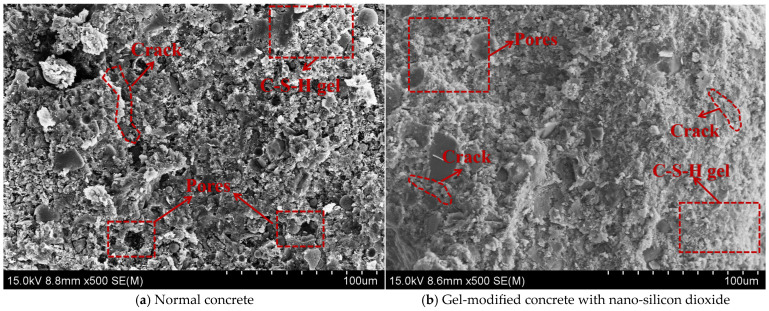
Concrete SEM results.

In this study, Nano-SiO_2_ supplied by McLean Reagent Company (Shanghai, China) was employed. The material appears as a white powder with a particle size of 20 ± 5 nm, a specific surface area of 193 m^2^/g, a PH of 6.9, and a purity of 99.5%. A visual representation of the nano-SiO_2_ powder is provided in Figure 12a. To ensure uniform dispersion, the nano-SiO_2_ suspensions were prepared using an HN-500 ultrasonic nanomaterial disperser, as illustrated in Figure 12b. Due to the addition of coal gangue, the water absorption of the aggregates is enhanced, consuming a large amount of free water, reducing the cement hydration products, and decreasing the pozzolanic effect of fly ash. As shown in Figure 13a, many unhydrated particles can be observed. After adding nano-silicon dioxide, due to its extremely high specific surface area, it rapidly generates the pozzolanic effect to produce C-S-H gel, and, at the same time, it effectively fills the large pores in the structure. Therefore, as shown in Figure 13b, a large amount of C-S-H gel with a high content is completely wrapped around the network fibre structure, and the structure is orderly stacked, forming a highly uniform continuous structure. Polypropylene fiber (PPF) obtained from Changsha Huixiang (Changsha, China) was also used, which is known for its cost-effectiveness and high shear strength. The fiber is supplied in the monofilament bundle form, with a specific gravity of 0.91, tensile strength exceeding 486 MPa, elastic modulus above 4.8 GPa, diameter ranging from 18 to 48 μm, and an elongation at break of 1%. The physical appearance of the PPF is shown in Figure 12c.

#### 4.1.4. Admixture

Polycarboxylic acid ether superplasticizer is used with a solid content of 30% (wt%) and a water reduction rate of 30% (wt%). The air-entraining agent used is YQ-2020 A sodium dodecyl sulfate (K12).

### 4.2. Mix Ratio Design and Specimen Preparation

#### 4.2.1. Concrete Mixture and Air-Entraining Agent Dosage

All concrete specimens were prepared with coal gangue as the only aggregate. The mix design featured a water-to-binder ratio of 0.48, a sand ratio of 0.45, 20% fly ash, and a 0.04% mass fraction of water-reducing agent. Nano-SiO_2_ (0.15% by mass) and PPF (0.6 kg/m^3^) were added with the aim of improving frost resistance by incorporating air-entraining agents at varying dosages (0%, 0.004%, 0.008%, 0.012%, and 0.016%). The specific amounts of nano-silica and PPF used were determined based on the literature [18]. This proportion guaranteed that the concrete’s fluidity met practical construction criteria. The complete mix design is summarized in Table 5.

#### 4.2.2. Preparation of Specimen

According to the Chinese standard GB/T50081-2019 [38], Nano-SiO_2_ was poured into half of the water, followed by ultrasonic dispersion. PPF was then added to the remaining half of the water, followed by stirring and dispersion. Next, the water-reducing agent and air-entraining agent were added to the PPF aqueous solution. After that, the blender was filled with cement, fly ash, and coarse coal gangue aggregate, and it was mixed for 90 s. Subsequently, the dispersed Nano-SiO_2_ solution was added to the mixer and stirred for 60 s. Afterwards, the PPF aqueous solution was added to the specimen and swirled for 90 s. For vibration moulding, the mixed concrete was placed into the moulds. The specimens were then cultured for 28 days in an incubator.

### 4.3. Experimental Method

In the experiment, the rapid freeze–thaw procedure was employed. The “Test Method for Long-term Performance and Durability of Ordinary Concrete” (GB/T50082-2009) standard [39] states that the minimum and maximum temperatures should be regulated at (−18 ± 2) °C and (5 ± 2) °C, respectively, and that the freeze–thaw cycle time should be set at 6–8 h. The test continued until it reached 100 cycles or one of the subsequent requirements was satisfied: (1) the specimen mass loss rate approached 5%; (2) the relative dynamic elastic modulus was lowered to 60%; and (3) strength loss surpassed 25%. The test method is shown as follows.

#### 4.3.1. Preparation of Freeze-Thaw Test Samples

The moisture on the surface of the sample from the freeze–thaw box was removed and the damage appearance damage of the sample was inspected. Through the freeze–thaw cycle test, concrete suffers different degrees of damage. Based on the comparison of the surface damage degree, the improvement degree of the different admixture dosages on the freeze resistance performance of coal gangue aggregate concrete can be judged.

#### 4.3.2. Mass Loss

After the appearance characteristics analysis of the concrete, the method for calculating the mass loss of the concrete specimens was tested by using the electronic balance. The calculation method is shown in Equation (5):

(5) $$W\;=\;\frac{G_{0}\;-\;G_{n}}{G_{0}}$$

where G_0_ is the concrete specimen’s initial mass, and G_n_ is the final mass after n freeze–thaw cycles. W is the concrete specimen’s mass loss rate after each freeze–thaw cycle.

#### 4.3.3. Mechanical Properties

**Figure 14:**
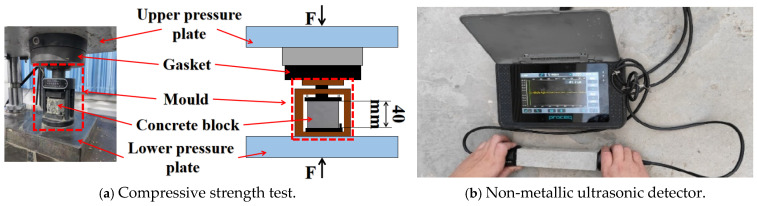
Test instrument.

The compressive strength test was performed on a cube mould with dimensions of 40 mm × 40 mm × 40 mm, as seen in Figure 14a. Three specimens from each group were tested to calculate the average result.

#### 4.3.4. Dynamic Modulus of Elasticity

The moisture on the surface of the test sample from the freeze–thaw box was removed and the damage appearance of the sample was examined. The dynamic elastic modulus of the sample was tested by using a non-metallic ultrasonic probe. The calculation method is shown in Equation (6):

(6) $$E_{\mathrm{RN}}\;=\;\frac{E_{\mathrm{dN}}}{\mathrm{Ed}0}=\frac{{\left(L/t_{N}\right)}^{2}}{{\left(L/t_{0}\right)}^{2}}=\frac{t_{N}^{2}}{t_{0}^{2}}\;\times \;100\%$$

where E_RN_ represents the specimen’s relative dynamic elastic modulus during freezing and thawing. The specimen’s initial dynamic elastic modulus is denoted by E_d0_(GPa). The specimens’ dynamic elastic modulus during a freeze–thaw is indicated by E_dN_(GPa). The specimen’s length, L(mm), is nearly constant both before and after freezing and thawing; t_0_(s) represents the ultrasonic wave’s propagation time in the specimen in its initial condition; and t_N_(s) represents the wave’s propagation time in the specimen following a freeze–thaw cycle [23].

#### 4.3.5. Capillary Water Absorption Test

**Figure 15:**
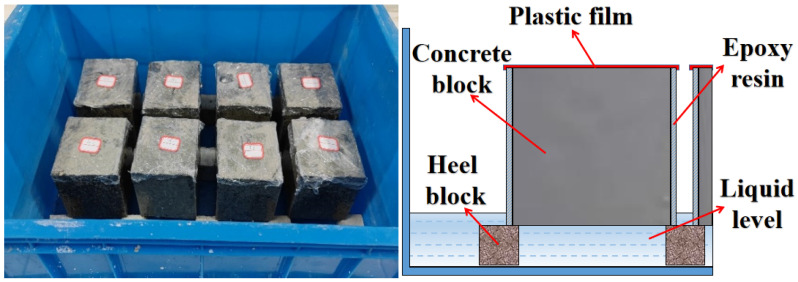
Concrete capillary water absorption test.

The ASTM C1585-13 [40] governs the capillary water absorption test for concrete. The specimen was dried to a constant weight (less than 0.1% mass loss in 24 h) at 60 °C before the test. Following the removal, the top surface was covered with plastic film to guarantee that only one surface comes into touch with water. The four sides were then coated with epoxy resin for hydrophobic treatment. As seen in Figure 15, the concrete specimen was submerged to a depth of 5 mm below the water’s surface while it was on the suction device’s pad during the test. The specimen’s mass was measured and noted under the time frame given in reference [29]. Table 6 displays the goal time and permitted errors for the measurement period. Water absorption and unit cumulative absorption may be derived using Equations (7) and (8).

(7) $$I=\frac{∆m}{A\times \rho }$$

(8) $$I=S\sqrt{t}+b$$

#### 4.3.6. Evaluation Method of Frost Resistance

The effect of the air-entraining agent content on the frost resistance of coal gangue aggregate concrete was evaluated using the grey relational degree technique. There are four stages to the data processing process. To begin, the reference sequence must be established. In this research, the reference sequence consists of the following parts: mass loss rate, relative elastic modulus, cumulative water absorption rate, initial water absorption rate, secondary water absorption rate, and later water absorption rate. The second step involves standardizing and non-dimensionalizing the original data. The correlation coefficient is then computed with the resolution coefficient (ρ) set to 0.5. The correlation degree is computed and compared in the last step. Comprehensive computation steps can be seen in reference [41]. Based on the degree of association, one may ascertain the extent to which various air entrainment dosages impact each of the seven concrete frost resistance indices. The impact element is more substantial the higher the degree of association.

## Figures and Tables

**Table 1:** Parameter fitting.

Test ID	A	B	R^2^
A0	0.05762	1.02738	0.99
A2	0.05594	1.02596	1.00
A4	0.05827	1.01900	0.99
A6	0.06002	1.01726	0.98
A8	0.06078	1.01335	0.98

**Table 2:** Comparison between the test value and the calculated value.

Type	Freeze–Thaw Cycles (Times)			
	0	25	50	75
Test value (TV)	0.058	0.087	0.157	0.237
Calculation value (CV)	0.058	0.096	0.160	0.267
TV/CV	1.00	0.90	0.98	0.89

**Table 3:** Chemical composition of cement and fly ash.

Compositions	Na_2_O	MgO	Al_2_O_3_	SiO_2_	SO_3_	K_2_O	CaO	Fe_2_O_3_	LOI
Cement	0.79	1.02	4.93	17.63	3.01	0.47	63.22	3.99	3.95
Fly ash	0.33	0.23	38.01	46.44	0.69	0.88	7.5	3.12	2.79

**Table 4:** Physical properties of coal gangue.

Coal Gangue	Bulk Density (kg/m^3^)	Apparent Density (kg/m^3^)	Water Absorption (%)	Porosity (%)	Moisture Content (%)	Crushing Value (%)
Coarse aggregate	1507	2740	6.9	45	2.0	22.4
Fine aggregate	1475	2620	2.7	44	0.9	10.2

**Table 5:** Concrete mix design (kg/m^3^).

Test ID (Air-Entraining Agent Dosage)	Cement	Fly Ash	Water	Fine Aggregate	Coarse Aggregate	Water Reducer	Nano-SiO_2_	PPF	Air-Entraining Agent
A0 (0%)	400	100	240	720	880	20	7.5	0.6	0
A2 (0.004%)	400	100	240	720	880	20	7.5	0.6	0.02
A4 (0.008%)	400	100	240	720	880	20	7.5	0.6	0.04
A6 (0.012%)	400	100	240	720	880	20	7.5	0.6	0.06
A8 (0.016%)	400	100	240	720	880	20	7.5	0.6	0.08

**Table 6:** Target time interval and allowable error.

Time	1 min	5 min	10 min	20 min	30 min
Tolerance	2 s	10 s	2 min	2 min	2 min
Time	60 min	One time per hour before 6 h	Once daily for up to 3 days	Once daily (Day 4 to 7)	One measurement (Day 8)
Tolerance	2 min	5 min	2 h	2 h	2 h

A is the water contact area (mm^2^), ρ is the water density (g/mm^3^), S is the water absorption (mmS^−0.5^), t is the water absorption period (s), and b is the curve deviation (mm). I is the specimen’s cumulative unit of water absorption, measured in millimetres.

## Data Availability

The data presented in this study are openly available in article.
